# Facial Reconstruction: A Systematic Review of Current Image Acquisition and Processing Techniques

**DOI:** 10.3389/fsurg.2020.537616

**Published:** 2020-12-07

**Authors:** Sam P. Tarassoli, Matthew E. Shield, Rhian S. Allen, Zita M. Jessop, Thomas D. Dobbs, Iain S. Whitaker

**Affiliations:** ^1^Reconstructive Surgery & Regenerative Medicine Research Group, Swansea University Medical School, Swansea, United Kingdom; ^2^Welsh Centre for Burns and Plastic Surgery, Morriston Hospital, Swansea, United Kingdom; ^3^College of Medicine, Swansea University Medical School, Swansea, United Kingdom

**Keywords:** reconstruction, facial 3D images, image acquisition, imaging technique, 3D printing

## Abstract

**Introduction:** Plastic and reconstructive surgery is based on a culmination of technological advances, diverse techniques, creative adaptations and strategic planning. 3D imaging is a modality that encompasses several of these criteria while encouraging the others. Imaging techniques used in facial imaging come in many different modalities and sub-modalities which is imperative for such a complex area of the body; there is a clear clinical need for hyper-specialized practice. However, with this complexity comes variability and thus there will always be an element of bias in the choices made for imaging techniques.

**Aims and Objectives:** The aim of this review is to systematically analyse the imaging techniques used in facial reconstruction and produce a comprehensive summary and comparison of imaging techniques currently available, including both traditional and novel methods.

**Methods:** The systematic search was performed on EMBASE, PubMed, Scopus, Web of Science and Cochrane reviews using keywords such as “image technique/acquisition/processing,” “3-Dimensional,” “Facial,” and “Reconstruction.” The PRISMA guidelines were used to carry out the systematic review. Studies were then subsequently collected and collated; followed by a screening and exclusion process with a final full-text review for further clarification in regard to the selection criteria. A risk of bias assessment was also carried out on each study systematically using the respective tool in relation to the study in question.

**Results:** From the initial 6,147 studies, 75 were deemed to fulfill all selection criteria and selected for meta-analysis. The majority of papers involved the use of computer tomography, though the use of magnetic resonance and handheld scanners using sonography have become more common in the field. The studies ranged in patient population, clinical indication. Seminal papers were highlighted within the group of papers for further analysis.

**Conclusions:** There are clearly many factors that affect the choice of image acquisition techniques and their potential at being ideal for a given role. Ultimately the surgical team's choice will guide much of the decision, but it is crucial to be aware of not just the diagnostic ability of such modalities, but their treatment possibilities as well.

## Introduction

The advancement of modern imaging techniques in plastic and reconstructive surgery has led to ever improving patient outcomes, allowing the surgeon to visualize and gain a better spatial appreciation during complex surgical procedures. Of those utilized, three-dimensional (3D) imaging has become commonplace due to its accuracy, precision, and versatility (1). 3D imaging has multiple modalities, each with their own advantages and disadvantages; these include, but are not limited to, magnetic resonance imaging (MRI), computed tomography (CT), 3D photography and handheld scanners using sonography (Table 1). All these modalities and their respective sub-modalities play their own role in the diagnosis, planning, and treatment of the patient, thereby, becoming a cornerstone of preoperative, perioperative, and postoperative care (2). In facial reconstructive surgery, the use of detailed facial imaging techniques is imperative for such an anatomically intricate area of the body; for this reason, there is a clear clinical need for hyperspecialized practice (3).

**Table 1:** Summary table highlighting the advantages and disadvantages of various imaging techniques.

**Imaging technique**	**Advantages**	**Disadvantages**
CT	• Provide detailed picture with tissue differentiation of both hard and soft tissues • Shows acute bleeds • Painless and noninvasive • Angiographic capabilities for specific preoperative planning	• Time consuming • Expensive (less than MRI) • Skilled technicians required • Ionizing radiation dose • Contrast materials may be required that can be harmful
MRI	• Provides a detailed picture of soft tissues structures more accurately than CT • No ionizing radiation dose • Painless and noninvasive • Contrast materials used are not as harmful • Angiographic capabilities for specific preoperative planning	• Time consuming • Expensive • Skilled technicians required • Not usable for patients with older surgical metals in body • Implantable medical devices may malfunction during use (i.e., pacemakers)
Handheld sonography scanners	• Portable, lightweight, and mobile—can be performed at the bedside • Quick and inexpensive • Adjunct to the clinical examination	• Lower resolution and image quality • Can be limited to 2D • Small screen size makes visualization difficult
2D photography	• Specialist training not required • Inexpensive • Easy and efficient downstream processing • Easily reproducible	• Does not provide detailed topographical measurements • Provides no subsurface imaging
3D photography	• Provides detailed topographical measurements of the face • Relatively inexpensive • Easily reproducible	• Specialist training required • Ease of availability

The advancement of facial reconstructive surgery has coincided with the evolution of the imaging modalities available for patient assessment, surgical planning, and postoperative follow-up. For decades, prior to the development of CT and MRI in the late 1970s, clinical 2D photography was utilized as the primary imaging modality for the objective measure of surgical outcomes (4). The use of 2D clinical photography continues to be a cornerstone of plastic surgery due to its availability, ease of obtainment, and reproducibility when re-examination is required (5).

The introduction of CT and MRI scans in the early 1980s as well as recent advancements in 3D photography and ultrasound scanners permit detailed geometric, topographical, and volumetric analysis, allowing the surgeon to form a more accurate clinical picture for preoperative surgical planning and/or intraoperative surgical guidance (6). Advancements in imaging modalities and increasing literature in this field necessitates a comprehensive review of the advantages and disadvantages of each imaging technique to act as a guide for clinicians involved in facial reconstruction. In addition to the high resolution of these imaging modalities, the ability to combine these with angiography components adds an extra and indispensable element to the preoperative planning process (7, 8).

With the variety of imaging techniques and computer processing systems available, identifying which case-specific imaging modality to use has become a challenge, with each modality having a situational-dependent advantage. The importance of an evidence-based imaging choice cannot be overstated when picking a modality; it becomes easy for bias to be formed through familiarity rather than best practice. Despite this, there is limited literature available that strives to provide conclusions and therefore limited guidance for surgeons on which imaging modality to utilize for specific facial reconstructive procedures.

Surgical planning is a broad term that encompasses several meanings; here, it refers specifically to the use of image acquisition and processing techniques used in the context of facial reconstructive plastic surgery. The aim of surgical planning is to simply acquire the most comprehensive representation of the structure being operating on, thus, visualizing the surgery about to be undertaken. This visualization can be done with single or multiple imaging techniques, often multimodal approaches providing the most comprehensive picture. Surgical planning begins with image capture of the specific anatomical area, these images are then rendered using modality-dependent processing techniques (9). The result of this is a virtual 3D model that allows for visualization of the anatomy from different angles and provides the surgeon with a “mental map” that allows for surgical navigation throughout the operation (10). With these tools at their fingertips, a clear stepwise approach can be taken for each operation. In addition to this, these walkthroughs can be shown to patients to allow education and inform the consent process and therefore improve patient outcomes (11).

The aim of this systematic review was to highlight all the available image acquisition and processing techniques that have been utilized for bony facial reconstruction in the literature. The goal is that this forms a comprehensive summary of the available bony facial imaging techniques for surgeons involved in facial reconstruction and guides decision making based on patient-specific needs.

## Methods

### Search Strategy

**Figure 1:**
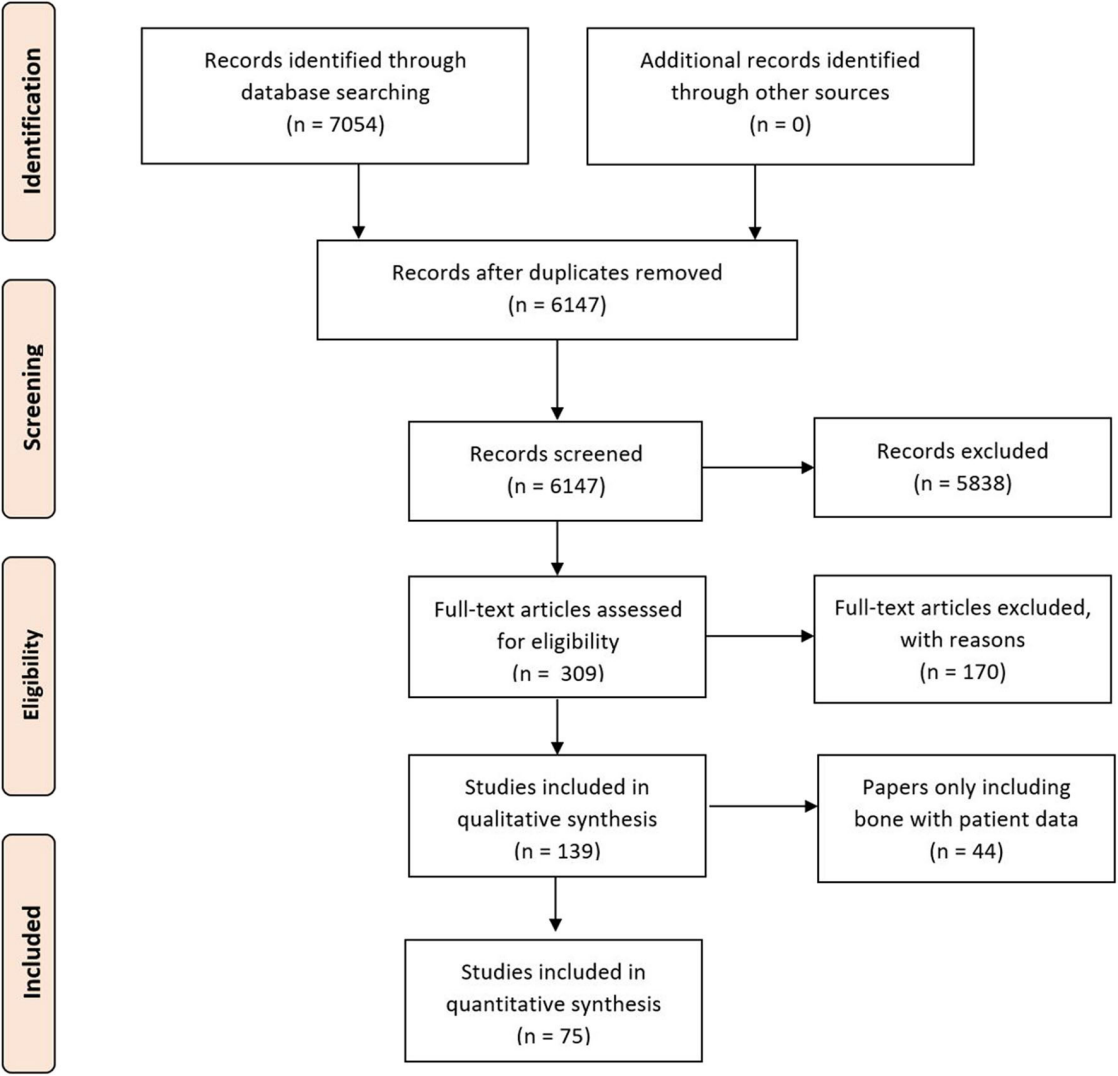
Preferred reporting items for systematic reviews and meta-analyses flow chart of included studies.

A systematic review protocol was developed in accordance with the recommendations of the Preferred Reporting Items for Systematic Reviews and Meta-Analyses (PRISMA) guidelines (Figure 1) (12) to evaluate the types of imaging techniques and their clinical variability in the literature To identify all relevant papers, a comprehensive search strategy was developed and pretested, with an example of the search strategy used; the full search having being provided later in the Methods section. Searches were performed in Web of Science (Web of Science Core Collection, BIOSIS Citation index, KCI-Korean Journal Database, MEDLINE, SciELO Citation Index), PubMed, Scopus, OVID, and Embase. The review was additionally registered on the International prospective register of systematic reviews (PROSPERO) (13).

The specific search and selection strategy performed is highlighted below and is based on the initial search strategy registered with PROSPERO. The keywords and strategy utilized is as follows;

“*(((facial OR maxillofacial) AND (reconstruction OR recon surgery)) AND ((image technique) OR (image acquisition) OR (image processing) OR ((computed tomography) OR (magnetic resonance imaging) OR (ultrasonography) OR (3D photography))))”*

This search strategy provided a clear but precise identification of relevant articles in the literature. These papers were then screened further by the use of specific eligibility and exclusion criteria.

#### Eligibility Criteria

Eligibility for article inclusion were (1) all studies included involved facial imaging techniques; (2) bone reconstruction; (3) included only imaging modalities aiming to visualize bony structures or imaging modalities aiming to visualize facial reconstruction; (4) clinical patient outcomes; (5) English language articles only.

#### Exclusion Criteria

Studies were excluded if they met the following criteria: (1) no clinical component; (2) involved non-facial surgical techniques; (3) purely soft tissue reconstruction; (4) contained no surgical component; (5) were not available for viewing. Review articles and commentaries were also excluded.

### Study Selection

Two reviewers (S. P. T. and M. E. S.) independently reviewed the studies with differences resolved by a third reviewer (R. S. A.). Titles were initially screened to exclude duplicates and further screened using the abstracts against inclusion and exclusion criteria. Finally, full text review of the remaining articles was performed to assess for eligibility.

### Data Extraction and Main Outcomes

Data were extracted from the selected studies using a standardized format (Microsoft Office Excel 2019). The initial tabulated data collection included: anatomy, patient demographic, study demographic, methodology, study design, primary and secondary outcomes, complications, and clinical availability.

### Risk-of-Bias Assessment

Studies were examined for bias using the Cochrane tool (RoB 2.0) (14) as a guide alongside modified versions of the tool and evaluations of the tool itself (15). The literature was separated into cohort-based (Table 2) and trial-based (Table 3) categories due to the differing criteria needed for assessing the bias (16).

**Table 2:** Risk-of-bias asssessment on cohort studies.

**References**	**Study design**	**Confounding**	**Selection of participants**	**Measurement of interventions**	**Departures from intended interventions**	**Missing data**	**Measurement of outcomes**	**Selection of reported results**	**Overall risk of bias**
An et al. (82)	Not stated								
Andrews et al. (31)	Multicenter retrospective								
Broumand et al. (22)	Not stated								
Davies et al. (55)	Retrospective cross-sectional								
Dong et al. (73)	Retrospective								
Eppley (24)	Clinical series								
Farook et al. (84)	Not stated								
Frellesen et al. (63)	Retrospective								
Fu et al. (51)	Retrospective								
Gander et al. (69)	Retrospective								
Gerbino et al. (28)	Case series								
Gibelli et al. (70)	Not stated								
Guest et al. (86)	Single-center case series								
Gui et al. (27)	Not stated								
Guo et al. (21)	Not stated								
Heiland et al. (74)	Not stated								
Heissler et al. (17)	Not stated								
Kim et al. (72)	Not stated								
Klenk and Kovacs (25)	Retrospective								
Kokosis et al. (85)	Retrospective								
Kraeima et al. (54)	Not stated								
Kwon et al. (64)	Not stated								
Lim et al. (80)	Not stated								
Liu et al. (33)	Not stated								
Mascha et al. (52)	Retrospective								
Myga-Porosiło et al. (79)	Not stated								
Novelli et al. (65)	Not stated								
Ohkawa et al. (58)	Not stated								
Rabie et al. (26)	Preliminary series								
Reiser et al. (57)	Not stated								
Sawh-Martinez et al. (53)	Not stated								
Schmutz et al. (29)	Cross sectional								
Shaye et al. (60)	Retrospective review								
Sozzi et al. (71)	Retrospective								
Suzuki et al. (40)	Not stated								
Suzuki-Okamura et al. (32)	Not stated								
Tabakovic et al. (67)	Not stated								
Taoet al. (59)	Not stated								
Tarsitano et al. (68)	Not stated								
Tarsitano et al. (61)	Not stated								
Tel et al. (36)	Not stated								
Tello et al. (23)	Not stated								
Thiele et al. (37)	Not stated								
Wang et al. (42)	Not stated								
Wang et al. (77)	Not stated								
Wang et al. (48)	Not stated								
Wilde et al. (81)	Retrospective								
Wu et al. (47)	Retrospective								
Xi et al. (43)									
Yu et al. (50)	Retrospective case series								
Yu et al. (45)	Not stated								
Zamora et al. (19)	Not stated								
Zamora et al. (18)	Not stated								
Zhang et al. (30)	Not stated								
Zhang et al. (56)	Retrospective review								
Zhang et al. (78)	Retrospective review								
Zheng et al. (62)	Not stated								
Zhou et al. (41)	Not stated								

*(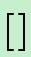
—low risk, 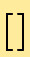
—moderate risk, 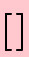
—high risk)*.

**Table 3:** Risk-of-bias asssessment on trial studies.

**References**	**Study design**	**Sequence generation**	**Allocation concealment**	**Blinding**	**Incomplete data**	**Selective reporting**	**Other sources**	**Overall risk of bias**
Ayoub et al. (44)	Randomized and prospective							
El-Fiky et al. (35)	Prospective							
Fan et al. (75)	Prospective							
Kolk et al. (76)	Prospective							
Schimming et al. (39)	Prospective							
Schimming et al. (38)	Prospective							
Shan et al. (46)	Prospective							
Tarsitano et al. (34)	Prospective							
Tenhagen et al. (20)	Prospective							
Tsao et al. (66)	Prospective							
Weijs et al. (49)	Prospective							

*(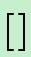
—low risk, 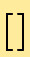
—moderate risk, 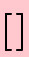
—high risk)*.

## Results

The initial study numbers that were found (after duplicate removal) were 6,147, which led to the final inclusion of 75. Papers were removed from the screening and data extraction stage if they did not abide by the criteria laid out in the PROSPERO protocol. The papers were categorized into the anatomical area of the face that the work correlated to and then arranged by the date of publication (Table 4).

**Table 4:** Imaging and printing techniques used in the studies.

**References**	**Study type**	**Imaging acquisition and technique**	**Printer**	**Number of patients**	**Mean Age**
**Cephalic**					
Heissler et al. (17)	Bony skull defects	Transversal 2-mm spiral CT with 3D reconstruction of the cranium were made.		15	27
Zamora et al. (18)	Cephalometric landmarks	Patients were selected who had both an LRC and a CBCT.		9	5.2
Zamora et al. (19)	Cephalometric landmarks	Scan using the “i- CAT” cone bean system		18	15.55
Tenhagen et al. (20)	Calvarial recon for scaphocephaly	3D handheld scanning photography (M4D Scan 3D scanner by Rodin4D, Vxelements software) vs. planar x-ray		9	5.2
Guo et al. (21)	Condylar head fracture preoperative planning	All patients underwent CBCT preoperatively, and Digital Imaging and Communications in medicine (DICOM) files were imported into Simplant 11.04 software.		13	41
**Craniofacial**					
Broumand et al. (22)	Craniofacial fractures and open reduction	CT scans performed on a General Electric 9,800 CT scanner		20	
Tello et al. (23)	Facial trauma recon	Patients were studied with either CCT or SCT of the face after trauma.		6	
Eppley (24)	Cranial or cranio-orbital recon	Preoperatively, a 3D CT scan (1-mm cuts) was obtained from which an anatomical model was fabricated. On the anatomical model, the predicted amount of bone excision was performed.	A tape of the scan was sent to the manufacturer and a 3D model generated.	7	32.7
Klenk and Kovacs (25)	Ct for facial fracture/bony pathology	Patients' radiographs and CT scans were reviewed to establish the clinical value of 3D CT.		121	
Rabie et al. (26)	Facial fractures	The xCAT ENT was used to provide images		3	
Gui et al. (27)	Craniofacial fibrous dysplasia recontouring	Preoperative and postoperative spiral CT data		21	23
Gerbino et al. (28)	Fronto-orbito-pterional craniotomy	Preoperative Spiral CT	Custom made prefabricated polyetheretherketone (PEEK) used to manufacture implants Surgical guides and implants produced using rapid prototyping technologies.	3	52
Schmutz et al. (29)	Imaging orbital fractures	MRI based virtual 3D models of the intact orbit		11	30
Zhang et al. (30)	Craniomaxillofacial bone defects	CT (slice thickness 0.625 mm), computer-aided design/computer-aided manufacturing and 3D reconstruction, as well as rapid prototyping were performed.	The customized HA/EAM compound artificial implants were manufactured through selective laser sintering using a rapid prototyping machine into the exact geometric shapes of the defect.	12	25.6
Andrews et al. (31)	Craniomaxillofacial surgery	All subjects undergo a fine cut noncontrast max- illofacial CT scan with 0.5- to 1-mm slice cuts.		20	42
Suzuki-Okamura et al. (32)	Le Fort 1 and sagittal split ramus osteotomies	3D CT images taken before and after surgery were superimposed by 3D imaging software.		9	24.6
Liu et al. (33)	Complex craniomaxillofacial surgery	Spiral CT data sets (light speed 16, General Electric, Fairfield, CT; 0.625-mm slice thickness) were acquired for all patients preoperatively.		15	26.9
Tarsitano et al. (34)	Disarticulation resection surgery for mandibular tumor, reconstructive plate supporting fibular microvascular free flap	Planning and postoperative CT scans were superimposed to assess the accuracy of reconstruction. Virtual planning began with acquisition of high-resolution CT scans of the craniofacial region and the lower legs (the donor site)		9	44
El-Fiky et al. (35)	Le Fort fracture imaging	All patients subjected to non-contrast MSCT in axial cuts. Multiplanar reformatted (MPR) images were acquired using the machine software in sagittal and coronal planes.		30	35.1
Tel et al. (36)	Craniofacial surgery	Preoperative CT, Digital Imaging and Communications (DICOM) files imported into Anatomage InVivo software for segmentation. Postoperative CT evaluated procedure accuracy.		3	
Thiele et al. (37)	Craniomaxillofacial recon	In all cases, CBCT datasets were uploaded online in DICOM format via a secured website. Implants were designed from these datasets using OsiriX and/or Mimics Medical 19.0 software. The titanium plates were 3D printed using the selective titanium laser sintering method.	Anatomical models were 3D printed using conventional 3D printers and sent to the hospital by post, along with the PSIs	51	60
**Mandibular**					
Schimming et al. (38)	Recon with microvascular bone graft	Computer-aided 3D reconstruction was performed according the following protocol: 15 min after injection planar scintigraphic images were acquired from frontal, dorsal, and lateral views.		20	
Schimming et al. (39)	Assess mandibular bone invasion (SCC)	Each patient was examined preoperatively clinically as well as by conventional radiography (panoramic radiography), CT scan and investigation. In all cases computer-aided 3D reconstruction of the acquired SPECT images were performed.		88	51.5
Suzuki et al. (40)	Cancer resection and reconstruction	Their radionuclide examinations and CT investigations were performed 1–83 days (median, 34 days) and 1–80 days (median, 30 days) before operation, respectively.		34	63
Zhou et al. (41)	Defect repair and autogenous bone graft	Spiral CT data acquisitions of the skulls were performed with a 1.25-mm slice thickness and a slice reconstruction interval of 0.625 mm	The tray was manufactured in the LPS 600 laser prototyping type of stereolithography system	6	28.5
Wang et al. (42)	Block resection mandible and recon with fibular flap	Based on the digital imaging and communications in medicine (DICOM) data from the CT	A physical resin model of the reconstructed mandible was manufactured using the SPS350 laser stereolithography prototyping system	10	29.1
Xi et al. (43)	Condyle recon	CBCT datasets were obtained by scanning the patients seated in the natural head position using a standard CBCT scanning		10	38.1
Ayoub et al. (44)	Reconstruction with iliac crest bone graft	Patients randomly allocated into two equal groups using the computer program RandList (DatInf GmbH, Tübingen, Germany). Virtual surgical planning was based on preoperative CT-data using specific surgical planning software [ProPlan CMF (Materialize NV, Leuven, Belgium)]. A rapid prototyping guide transferred the virtual surgery plan to the operation site. To compare pre and postoperative condyle position, intercondylar distance was measured using 3D models of the mandible before and after surgery. Models were imported into the Geomagic Studio software (Geoma- gic, Morrisville, NC, USA) using the STL-format.		20	53
Yu et al. (45)	Condylar resection and condylectomy	A preoperative thin-cut (1.25 mm), spiral CT scan was obtained for all patients.		5	25.4
Guo et al. (21)	Condylar head fracture preoperative planning	All patients underwent CBCT preoperatively, and Digital Imaging and Communications in medicine (DICOM) files were imported into Simplant 11.04 software.		13	41
Shan et al. (46)	Reconstruction with fibula flap	Computed tomography (CT) scan, preoperative design, and operation on the mandible were done.		20	33
Wu et al. (47)	Reconstruction with fibula free flap	Computed tomography (CT) scanning was performed using a 64-slice CT unit. The CT data of the skull and the fibula were transferred to the ProPlan CMF 1.4 software.		8	32.6
Wang et al. (48)	Reconstruction with vascularized fibula graft	ProPlan CMF surgical planning software		56	52
Weijs et al. (49)	Segmental resection (with fibular free flap - not evaluated)	Preoperatively, a CBCT scan was acquired to delineate the size and extension of tumor invasion; patients in natural head position, using a standard CBCT scanning protocol.		11	68
Yu et al. (50)	Mandibulectomy and mandibular recon with free fibula flap	The process of CAD began with the acquisition of high-resolution CT scans of the maxillofacial skeleton and lower extremities. The imaging and planning plat- form used in this study was Surgicase CMF		29	33
Fu et al. (51)	Contour surgery	Perform VSP based on 3D computed tomography (CT) data.		20	25.4
Mascha et al. (52)	Reconstruction with vascularized and non-vascular bone graft	Mandibular reconstruction with the PSMP-method. Preoperative and postoperative CT scans were evaluated by measuring distances between corresponding landmarks on the mandibular rami. The difference was used to evaluate reconstruction accuracy.		18	65
Sawh-Martinez et al. (53)	Reconstruction of TMJ position	Preoperative CT of the mandible in all patients		16	61.6
Kraeima et al. (54)	Reconstruction	Each patient underwent diagnostic work-up consisting of both a CT and MRI of the head and neck region according to the clinical protocol.		34	69.9
Davies et al. (55)	Reconstruction	High-resolution CT scans were acquired using multidetector CT with standard protocols exhibiting nearly isotropic 3D spatial resolution for the facial bones. The image voxel size was 0.47 mm^3^.		10	55
Zhang et al. (56)	Reconstruction with iliac crest flap	All patients consented to undergo 3D CT and image reconstruction, mirror imaging design, 3D model prototyping, CTA, fabrication of an individual preformed reconstruction plate, and iliac crest flap design before surgery.		19	15.8
Reiser et al. (57)	Oromandibular recon (virtual resection and free fib flap)	CT was obtained	V-stand is 3D printed using biocompatible plastic polymers.	17	53
**Maxillary surgery**					
Ohkawa et al. (58)	Maxillofacial fractures	2D CT and 3D CT with helical CT scanning were performed using Toshiba Xvigor scanner.		21	
Tao et al. (59)	Maxillary and mandibular tumors	In this study, the maxillofacial tumors were subjected to a mimic operation on a computer following CT scanning and 3D reconstruction.		10	45
Shaye et al. (60)	Maxillofacial recon	Intraoperative CT scans were obtained for all patients.		38	37.4
Tarsitano et al. (61)	Maxillary recon with fibular flap	Preoperative high-resolution CT data set used for virtual planning was superimposed onto the postoperative CT	Reconstructive titanium mesh was manufactured by a direct metal laser sintering (DMLS) method. The solid-to-layer files of the guide and plate were then manufactured by DMLS using an EOSINT M270 system	4	
Zheng et al. (62)	Maxillary reconstruction	CT data processed using Mimics 10.01 software	3D printer to print all templates.	6	35.6
Frellesen et al. (63)	Maxillofacial trauma	Second-generation DSCT		120	29.975
**Orbital and zygomatic**					
Kwon et al. (64)	Orbital blowout fractures	Facial CT scans before and after surgery.		24	33.2
Novelli et al. (65)	Orbital recon	DICOM data was captured with a maxillofacial CT scanner that produces 0.8e1 mm slices. The CT was acquired after positioning the patient's landmarks in order to orient the patient in space during surgical navigation. Stereolithographic model was manufactured by exporting the patient's STL file of the skull and of the maxillofacial regions.	The STL model was printed by ZPrinter 310 (a rapid prototyping machine) through an additive technique using deposition of chalk	11	32
Tsao et al. (66)	Orbital wall fracture and recon with bone graft	Orbital reconstruction with radiopaque grafts (bone, titanium-reinforced polyethylene, and titanium plate) and assessed postoperatively with orbital CBCT (CS 9300; Carestream Health Inc., Rochester, NY).		4	49.5
Tabakovic et al. (67)	Orbital floor blowout fractures	Waters occipitomental view x-ray, 3D		10	30
Tarsitano et al. (68)	Recon orbital floor fracture	High-resolution CT scan of the patient's craniofacial skeleton. Imaging was performed using a multidetector CT scanner The solid-to-layer files of the mesh were then manufactured using direct metal laser sintering, which resolves the shaping and bending biases inherent in the indirect method.		7	
Gander et al. (69)	Zygomatic fracture	Preoperative multislice CT Intraoperative 3D CBCT		48	53.04
Gibelli et al. (70)	Normal zygomatic bone imaging	3D models of the zygomatic bone acquired through segmentation on CT scans		100	45.3
Sozzi et al. (71)	Orbital wall recon for craniofacial trauma	Reconstructed orbits from patients and control subjects were segmented from the postoperative CT scans. Postoperative CT scan 1 day (0–2 days) after reconstructive surgery using a 16-slice CT (Brilliance® Philips, Milan, Italy) with 2-mm thickness, 1mm increment acquisition, 1.5-mm thickness, 0.75-mm increment images reconstruction.		20	41.6
Kim et al. (72)	Orbital fracture	Pseudoforamina of the orbital wall were offset with the segmented sinuses. Finally, the 3D facial bone model, with orbital wall, was reconstructed from the segmented images. The CT data sets comprised slice images ranging from 171 slices to 246 slices.		10	45
Dong et al. (73)	Orbital wall fracture recon	The HA/PLLA implant was delivered in the form of a composite sheet, 0.3 or 0.5 mm in thickness, and a bone fixation tack system. Specifically, the preoperative CT images were imported into the workstation while the camera was pointed at the anticipated surgical site.		10	57.5
**Complex surgeries**					
Heiland et al. (74)	Zygomaticomaxillary complex fracture	Intraoperatively, after open reduction, a cone-beam CT (CBCT) dataset was generated using the SIREMOBIL Iso-C3D		14	43.9
Fan et al. (75)	Complex orbital fracture	CAD/CAM technique based on Helical CT	Porous polyethylene materials were shaped and inserted into the orbit to repair the orbital wall defect and correct the enophthalmos.	17	32.2
Kolk et al. (76)	Complex orbital recon	MSCT (Somatom Volume Zoom scanner, Sensation 16, Siemens Medical Solutions, Erlangen, Germany) and MR images as well as corresponding 3D reconstructions were used to assess the site and size of bony and soft tissue changes in the traumatized orbits.		37	30.6
Wang et al. (77)	Recontouring of craniomaxillofacial fibrous dysplasia	Preoperative thin-cut (0.625 mm), spiral CT scans were obtained.		13	27.3
Zhang et al. (78)	TMJ replacement surgery	3D CT scanning with a 16-spiral imager (0.625-mm slice thickness; LightSpeed Ultra; General Electric, Milwaukee, WI) of the craniofacial skeleton. The data from CT scanning in DICOM (Digital Imaging and Communications in Medicine) format were input into the interactive Simplant CMF software program (Materialize Medical, Leuven, Belgium). Preoperative planning included segmentation and osteotomies. The movements of the jaw bones were simulated by use of Simplant CMF. The affected mandible was reconstructed based on the contralateral side. The titanium plate was shaped on the reconstructed model before surgery. The bone graft was transplanted by the shaped titanium plate during the operation to reconstruct the TMJ.		11	42.3
Myga-Porosiło et al. (79)	Traumatic facial fractures	CT with a “Hispeed” unit		67	35
Lim et al. (80)	Temporal bone fracture (and facial nerve paralysis)	A high-resolution CT scan of the temporal bone was obtained in 1-mm sections with a CT scanner.		12	36.7
Wilde et al. (81)	Zygomatico-orbital complex fracture repair	Preoperative MSCT by use of multiplanar view reduction and internal fixation through an intraoral maxillary vestibular approach. Intraoperative 3D C-arm imaging		21	44
An et al. (82)	Resection of orbital craniofacial fibrous dysplasia	A preoperative 3D CT examination After CT scanning, the skulls were reconstructed in 3D using analytic software.		5	22.6
Shan et al. (83)	Maxillary and mandibular reconstruction	High-resolution CT of maxillofacial and fibula regions.		4	
Farook et al. (84)	Traumatic cranial and facial fractures	Patients were scanned in a Philips Ingenuity Core 128 Slice CT Machine with appropriate brain and facial protocols with bone and soft tissue reconstruction.		100	32
Kokosis et al. (85)	Complex mandibular recon	The initial CT scans were reviewed by our team, and VSP was undertaken using specialized software		5	
Guest et al. (86)	Skull components	A 3D model was produced for each of the seven participating patients based on preoperative cross-sectional imaging.	STL files were printed using a Stratasys uPrint SE Plus.	7	30.7

*CBCT, cone-bean computed tomography; CCT, conventional computed tomography; CT, computed tomography; CTA, computed tomography angiography; DSCT, dual-source computed tomography; HA/EAM, hydroxyapatite combined with epoxide acrylate maleic; HA/PLLE - hydroxyapatite combined with poly-l-lactide; LRC, locoregional treatment; MSCT, multislice computed tomography; PSMP, patient-specific mandible reconstruction plates; SCT, spiral computed tomography; SPECT, single photon emission computed tomography; VSP, virtual surgical planning*.

**Figure 2:**
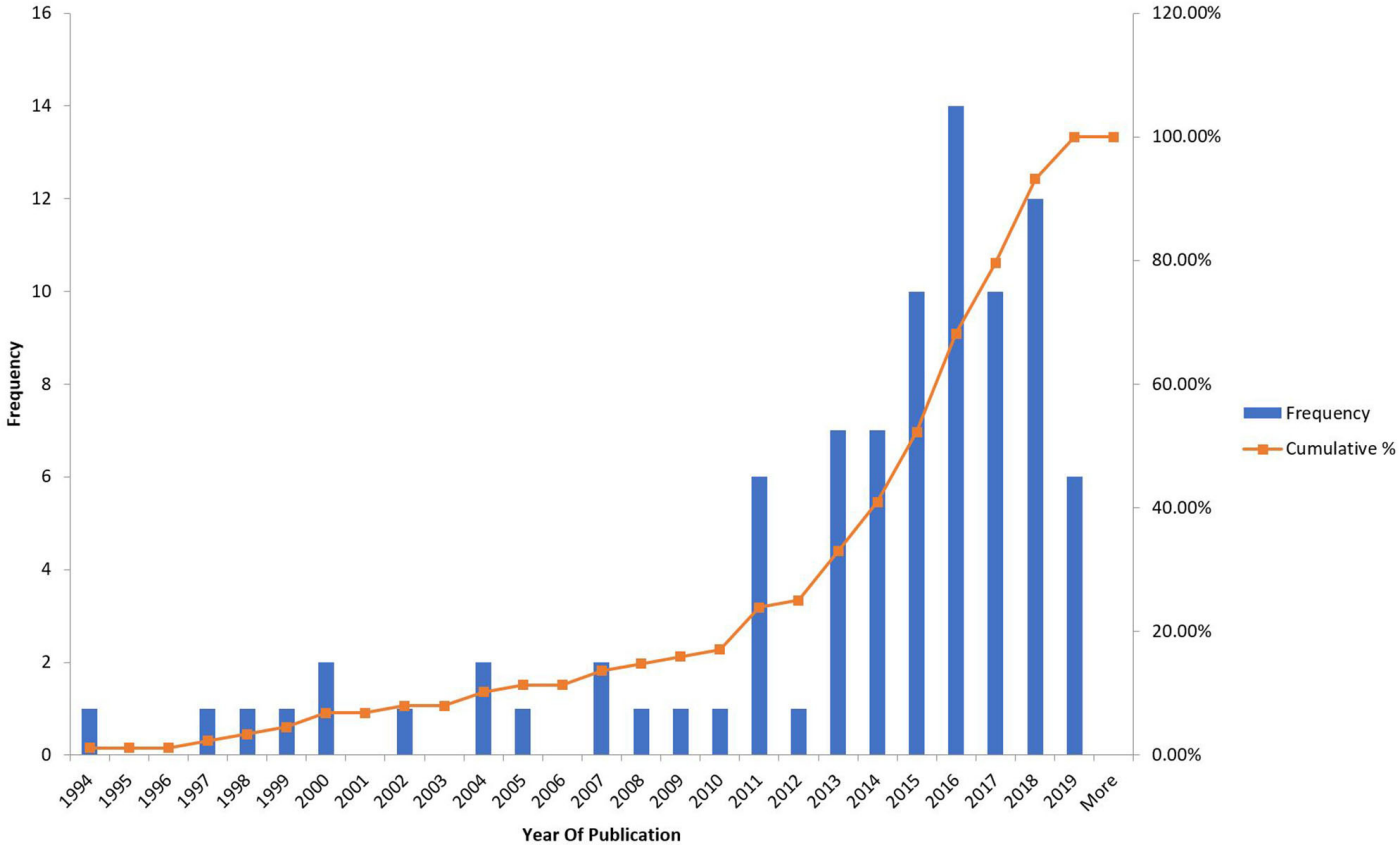
Frequency and cumulative count of studies by year of publication.

The frequency of the studies was looked at depending on the year of publication (Figure 2) and it was found that there was an almost exponential rise after 2011 which would be explained by the readiness of the technology available and the emergence of 3d printing abilities.

Risk-of-bias assessments were carried out using different tools that depended upon the type of study that was being assessed, i.e., cohort vs. trial-based study.

## Discussion

As can be seen from the analysis and results collation, there are many varying papers in the literature that use facial imaging in the clinical environment. Whether for diagnosis, treatment or prognosis, these techniques are commonplace in surgical theaters throughout the world and will become even more so in the future. It becomes a difficult task to analyze all the studies in a quantifiable sense due to the sheer variability between them, but it would be prudent to highlight what could be seminal papers in the field so that they can be turned to as reference. Not only are the key points of the papers crucial, but the impact factor of the journal and the ideals that they hope to achieve in future studies.

The seminal papers for the imaging modalities discussed have been selected through multiple means following a rigorous search strategy and certain criteria. These include but are not limited to: the papers impact factor, cohort study size, study type and low bias. The seminality of the papers selected has been based on the authors being the first to report specific imaging modality technique or being the first to utilize it in clinical practice with reportable and significant current and or future clinical impact.

The limitations of the search and the analysis of data should be noted. With such a large volume of papers to analyze there will be certain branches of facial surgery that were excluded. The foremost being orthognathic surgery. It was considered to include this in the search and analysis of papers, but it was decided that due to the complexity and the vastness of the topic (as it stetches into the world of maxillofacial and dental medicine) to not include these papers. Though one cannot argue the benefit for including these papers in the global scheme of analysis of facial imaging techniques, but with the search that was used, this would not be thorough or robust enough to include the seminal orthognathic papers (87). Similar to this, the use of alloplastic materials are a potential limitation of the review and search strategy. Though the systematic review did not intend to specifically mention imaging of alloplastic implants, we are aware of this challenge and the clinical benefit of the techniques used (88). However, further research would be needed to explicitly look at these criteria. Another limitation of this study is that though a comparable analysis of papers was attempted to be made with data extraction, due to the heterogeneity of the data and the techniques themselves, a true meta-analysis could not be carried out. However, the variability of clinical cases shows that perhaps a meta-analysis would not be of great benefit as it will not provide data that would be translatable to practice.

A detailed and comprehensive research strategy allowed for the exclusion of many papers. Where impact factor was low or cohort size restricted to minimal patients, there was a greater likelihood of exclusion. Although, it should be noted that low impact factor or cohort size did not guarantee exclusion if, in fact, that paper was deemed “seminal” in nature due to the technique utilized or significance of the study.

### Computed Tomography Scanning Systems

1. Wilde et al. Intraoperative imaging with a 3D C-arm system after zygomatico-orbital complex fracture reduction (81)

**Impact Factor 5.38**.

#### Intraoperative 3D CT C-Arm System

The purpose was to investigate whether intraoperative 3D C-arm imaging is effective technique for assessing the adequacy of fracture reduction in the management of uncomplicated ZMC fractures. They found that the use of 3D CT to assess fracture reduction intraoperatively shows to be an effective tool for evaluating ZMC fracture reduction. Therefore, avoiding additional procedures or need for further imaging.

Bias limitations: Moderate risk of bias from the measurement of interventions (retrospective review).

2. Ayoub et al. Evaluation of computer-assisted mandibular reconstruction with vascularized iliac crest bone graft compared to conventional surgery: a randomized prospective clinical trial (44)

**Impact factor 5.29**.

#### Computer Assisted vs. Conventional Surgery

Virtual surgical planning was based on preoperative CT-data using specific surgical planning software. A rapid prototyping guide transferred the virtual surgery plan to the operation site.

The purpose/aim of this study was to evaluate the benefits of computer-assisted mandibular reconstruction with iliac crest bone grafts. It compared the following areas:

- Intraoperative time for transplant shaping
- Ischemia
- Duration of surgery
- Amount of bone removed
- Change in postoperative condyle position compared to conventional surgery.

This is the first randomized prospective study comparing computer-assisted mandibular reconstruction with conventional surgery using iliac crest bone grafts.

The study shows that computer-assisted reconstruction reduces ischemic time and transplant-shaping time at the defect site as well as requiring a smaller amount of harvested bone than conventional surgery. Moreover, a significantly smaller alteration in condyle position could be shown in the computer-assisted group.

Bias limitations: High risk of bias from the blinding technique (trial format study).

3. Heiland et al. Intraoperative imaging of zygomaticomaxillary complex fractures using a 3D C-arm system (74)

**Impact factor 3.69**.

#### Intraoperative 3D CT C-Arm System

Purpose/aim of the study was to investigate in a first series patients with ZMC fractures, the practicability and value of intraoperative imaging using the 3D CT C-arm (SIREMOBIL Iso-C3D) after open reduction.

They showed that this interoperative imaging technique provided suitable visualization of the facial skeleton postoperatively. This study was done in 2004; therefore, image processing took longer to generate and would not be a technical limitation if carried out now. This paper has similar ideals to Wilde et al. but is important to mention as it paved the way for much of the current modern work.

Bias limitations: Moderate risk of bias from the measurement of interventions (retrospective review).

4. Zhou et al. Accurate reconstruction of discontinuous mandible using a reverse engineering/computer-aided design/rapid prototyping technique: a preliminary clinical study (41)

**Impact factor 5.09**.

#### Reverse Engineering (RE), Computer-Aided Design (CAD), and Rapid Prototyping (RP)

The aim/purpose of the study was to improve surgical outcomes in discontinuous mandibular defects by fabricating patient-specific customized titanium mandibular trays. The was done by utilizing reverse engineering (RE), computer-aided design (CAD) and rapid prototyping (RP) techniques by firstly obtaining a virtual 3D model via spiral CT scanning - the opposite side of the mandible was mirrored to cover the defect area to restore excellent facial symmetry. They then used autogenous bone grafting to restore the bony continuity for occlusion rehabilitation.

The trays fabricated using this technique fitted well in all six patients. The reconstructive procedures were easy and time saving. Satisfactory facial symmetry was restored. No severe complications occurred in the five patients without occlusion rehabilitation during a mean 50-month follow-up period. The reconstruction in the patient with occlusion lasted for only 1 year and failed eventually because of bone resorption and infection.

Satisfactory aesthetic results were achieved. However, the rigidity of the cast tray could cause severe stress shielding to the grafts, which could lead to disuse atrophy. A promising technique but some modification is needed for functional reconstruction.

Bias limitations: Moderate risk of bias from the selection of participants (retrospective review).

5. Shaye et al. Use of intraoperative computed tomography for maxillofacial reconstructive surgery (60)

**Impact factor 3.67**.

#### Intraoperative 3D CT C-Arm System

Aim/purpose of the study is to evaluate the time needed to perform intraoperative CT scans during maxillofacial surgery. In addition, they looked to see if there were any trends toward shorter total scan times as experience is gained with the technique. They also looked to identify the characteristics of cases that required intraoperative revision based on the results of intraoperative CT scanning.

##### Conclusions

- Current intraoperative CT scanning techniques are rapid, averaging 14.5 min per case (in 38 cases).
- No decrease in total scan time was noted during the study; however, the surgeon most experienced with the CT software had the shortest total scan times.
- Intraoperative revisions were most common in complex cases.

Recommendation from the study is that surgeons consider the use of intraoperative CT imaging for maxillofacial reconstruction, particularly in complex procedures.

Bias limitations: High risk of bias from the measurement of outcomes and selection of reported results (retrospective review).

6. Wang et al. Mandibular reconstruction with the vascularized fibula flap: comparison of virtual planning surgery and conventional surgery (48)

**Impact factor 5.6**.

#### Virtual Planning vs. Conventional Surgery

The aim/purpose of this study was to evaluate the accuracy of mandibular reconstruction and assessed clinical outcomes in both virtual planning and conventional surgery patients.

The main outcomes measured:

- Operative time
- Ischemia time
- Postoperative CT scans
- Facial appearance and occlusal function.

The ischemia time and total operation time were shorter in the virtual planning group than in the conventional surgery group.

High precision with the use of the cutting guides and templates was found for both the fibula and mandible, and a good fit was noted among the pre-bent plate, mandible, and fibula segments in the virtual planning group. Postoperative CT scans also showed excellent mandibular contours of the fibula flaps in accordance with virtual plans in the virtual planning group.

Surgical planning software (ProPlan CMF) was used preoperatively in the virtual planning group. In the virtual planning group, fibula flaps were harvested and osteotomized, and the mandibles were resected and reconstructed assisted by the prefabricated cutting guides and templates.

This study demonstrated that virtual surgical planning was able to achieve more accurate mandibular reconstruction than conventional surgery. The use of prefabricated cutting guides and plates makes fibula flap molding and placement easier, minimizes the operating time, and improves clinical outcomes.

Bias limitations: Medium risk of bias from the selection of participants, missing data, measurement of outcomes, and selection of reported results (retrospective review).

7. Wang et al. Three-dimensional virtual technology in reconstruction of mandibular defect including condyle using double-barrel vascularized fibula flap (42)

**Impact factor 5.38**.

#### 3D Virtual Planning

The aim/purpose of this study is to look at the impact of using 3D virtual surgical planning technology on surgical outcomes in the reconstruction of mandibular defects—specifically, type H mandibular defects including the condyle using a double-barrel vascularized fibula flap.

The simulation allowed for the construction of an individual mandibular model serving to guide the clinical operation. They found preoperative virtual surgery greatly benefitted the actual surgery. In addition, postoperative 3D reconstruction revealed a close match with the simulated condyle. Therefore, combined virtual 3D reconstruction and rapid prototyping can improve postoperative outcomes in mandibular reconstruction.

Bias limitations: Medium risk of bias from the selection of participants, missing data, measurement of outcomes, and selection of reported results (retrospective review).

8. Zhang et al. Evaluation of alveolar bone grafting using limited cone beam computed tomography (56)

**Impact factor 3.11**.

#### Limited Cone Beam CT (LCBCT)

The aim/purpose of the study is to look at the use of limited CBCT and subsequent 3D reconstruction to monitor bone resorption in alveolar bone grafts in patients with an alveolar cleft.

They found that LCBCT scan and 3D reconstruction is a promising method for evaluation of the outcome of alveolar bone grafts.

One of the first and only uses of limited cone beam and grafting combination.

Bias limitations: Medium risk of bias from the measurement of interventions and departure from the intended interventions (retrospective review).

9. Heissler et al. Custom-made cast titanium implants produced with CAD/CAM for the reconstruction of cranium defects (17)

**Impact factor 3.87**.

#### 3D Spiral CT With CAD/CAM to Produce Custom-Made Titanium Implants

The aim/purpose of this paper was to use 3D spiral CT to design titanium implants for bony skull defects. They used rapid prototyping for a fine casting process—the first group to do this.

One of the first papers to use spiral CT with CAD/CAM to create custom-made cast titanium implants for the reconstruction of cranium defects. They showed that this method is superior for several reasons for the patient and the surgeon:

- Complex geometrical structures with small diameters can be produced with significantly more precision than previously.
- The rim of the implant can be designed in such a way that it extends over the edge of the bone - this leads to good stability at the bone-implant interface.
- The results are predictable and aesthetically very pleasing.
- Operating time and the trauma caused by the operation are considerably less compared with techniques using autologous bone

Bias limitations: Medium risk of bias from the selection of participants, missing data, measurement of outcomes, and selection of reported results (retrospective review).

10. Yu et al. Three-Dimensional Accuracy of Virtual Planning and Surgical Navigation for Mandibular Reconstruction with Free Fibula Flap (50)

**Impact factor 3.2**.

#### CAD Surgical Navigation

The aim/purpose of the paper was to see if the use of CAD and surgical navigation improved the surgical outcomes and operations in free fibula flap mandible reconstruction for patients with benign tumors who underwent primary unilateral reconstruction.

They found that when including computer-aided design (CAD), CAD guided mandibular angle remodeling and condyle placement with increased accuracy. In addition, CAD and surgical navigation increase reconstruction accuracy without prolonging operative time.

Bias limitations: No risk of bias limitations (retrospective review).

### Magnetic Resonance and Handheld Scanning Imaging Modalities

1. Schmutz et al. Magnetic resonance imaging: an accurate, radiation-free, alternative to computed tomography for the primary imaging and three-dimensional reconstruction of the bony orbit (29)

**Impact factor 2**.

#### MRI vs. CT

The aim/purpose of the study is to compare the accuracy of MRI based virtual 3D models of the intact orbit can approach that of the gold standard, CT-based models. This is to identify whether MRI is a viable alternative to CT scans in patients with isolated orbital fractures and penetrating eye injuries, pediatric patients, and patients requiring multiple scans in whom radiation exposure is ideally limited.

Patients who presented with unilateral orbital fractures to a hospital in a 1-year period were recruited. The outcome measurements were orbital volume (primary outcome) and geometric intra-orbital surface deviations (secondary outcome) between the MRI- and CT-based 3D models.

The volumetric differences of the MRI models are comparable to reported results from CT models. The intra-orbital MRI surface deviations are smaller than the accepted tolerance for orbital surgical reconstructions. Therefore, the authors believe that MRI is an accurate radiation-free alternative to CT for the primary imaging and 3D reconstruction of the bony orbit.

Bias limitations: High risk of bias from the selection of participants, missing data, and measurement of outcomes (retrospective review).

2. Tenhagen et al. Three-Dimensional Handheld Scanning to Quantify Head-Shape Changes in Spring-Assisted Surgery for Sagittal Craniosynostosis (20)

**Impact factor 2.8**.

#### 3d Handheld Scanning and Postprocessing Imaging Techniques

The aim/purpose of the study was to assess the utility of 3D handheld scanning photography in a patient group which underwent spring-assisted correction surgery for scaphocephaly. Usually, the gold standard for acquiring 3D imaging is CT that entails ionizing radiations and, in young children, a general anesthesia. 3D photographic imaging is an alternative method to assess patients who have undergone calvarial reconstructive surgery.

They did Pre- and postop 3D scans acquired in theater, they then repeated these at the 3-week follow-up in clinic; images were then postprocessed for nine patients.

The following parameters were looked at:

- Cephalic index (CI)
- Head circumference
- Volume
- Sagittal length
- Coronal width over the head

Statistical shape modeling (SSM) was used to calculate the 3D mean anatomical head shape, no significant differences were observed in the CI between 3D and x-ray. Therefore, 3D handheld scanning followed by SSM proved to be an efficacious and practical method to evaluate 3D shape outcomes after spring-assisted cranioplasty in individual patients and the population—so a good alternative (or at least as a follow up) to CT.

Bias limitations: Medium risk of bias from the sequence generation, allocation concealment, and blinding (trial format study).

## Conclusion

The field of facial imaging techniques for bony reconstruction can be seen to be clearly computed tomography heavy, but some small gains have been made in the magnetic resonance and handheld field.

One could think that with such a large analysis of the literature, it could serve as a guide for those looking to plan preoperatively. However, this would merely be sweeping generalizations of the technologies available. There are several things that are clear and should help when planning surgical intervention. The use of CT is the most commonly used modality for bony injuries and defects due to its availability, versatility (with modifications such as angiography) and accuracy. The emergence of technologies such as CAD lend themselves to using CT and provide an extra level of preoperative data. Similar to CT, MRI has many of these of these benefits, but for purely bone based planning it would not be the modality of choice. Depending on the situation of the patient (such as renal issues), MRI may end up being the image technique of choice and luckily it is robust enough to be of benefit. To make full use of the analysis carried out in this work, one should attempt to tailor their case to those papers with similar patient populations and intended outcomes.

The future of the technology is still unclear as it will rely on certain surgical technological milestones to be met (quicker image processing techniques and smaller devices) alongside user preferences. However, there is an ultimate patient benefit to being able to pre-plan procedures in both outcomes and time. There are still costs related to this and these techniques may be difficult to integrate into developing countries and centers without the equipment or user ability on site.

## Data Availability Statement

The datasets generated for this study are available on request to the corresponding author.

## Author Contributions

ST: wrote majority and put paper together. MS: wrote parts of discussion and majority of introduction. RA: data extraction. ZJ and TD: data analysis and draft work. IW: oversaw project and draft work. All authors contributed to the article and approved the submitted version.

## Conflict of Interest

The authors declare that the research was conducted in the absence of any commercial or financial relationships that could be construed as a potential conflict of interest.
